# Incidental Diaphragmatic Hernia Discovered During Laparoscopic Sleeve Gastrectomy and Cholecystectomy: A Case Report

**DOI:** 10.7759/cureus.97389

**Published:** 2025-11-20

**Authors:** Haytham Mohammed Alzinati, Fauwaz F Alrashed, Abdullah Mustafa, Abdulmalek Ayman Arbach, Shahad Yaser Mustafa, Ahmed El Khodary, Zainab Hamed, Hanin Almorjanah

**Affiliations:** 1 General Surgery, Saudi German Hospital Hail, Hail, SAU; 2 Surgery, Bolu Abant Izzet Baysal University, Bolu, TUR; 3 General Surgery, Ibn Sina University, Khartoum, SDN; 4 General Surgery, Sulaiman Al Rajhi University, Al-Bukayriyah, SAU; 5 Surgery, Alexandria University, Alexandria , EGY; 6 Surgery, Elrazi University, Khartoum, SDN

**Keywords:** bariatric surgery, congential diaphragmatic hernia, conventional laparoscopic cholecystectomy, incidental surgical finding, laparoscopy, patient consent

## Abstract

Diaphragmatic hernias in adults are uncommon, especially when discovered incidentally during surgery for unrelated abdominal pathology. Incidental findings during bariatric surgery, such as diaphragmatic hernias, pose challenges in intraoperative decision-making. We report a rare case of a large left-sided diaphragmatic hernia discovered during laparoscopic sleeve gastrectomy. A 34-year-old male patient with morbid obesity (BMI 45.17 kg/m²), chronic calculous cholecystitis, and right hypochondrial pain underwent planned laparoscopic sleeve gastrectomy and cholecystectomy. Intraoperatively, a large left diaphragmatic hernia containing colon and small bowel was identified, but laparoscopic sleeve gastrectomy was aborted due to a lack of consent for hernia repair and the complexity of the concurrent procedure. Cholecystectomy proceeded uneventfully, with histopathology confirming chronic cholecystitis. Postoperative recovery was smooth, with a pending management plan for the unrepaired hernia. This case illustrates the importance of thorough preoperative imaging, anticipation of possible anatomic surprises, and sufficiently broad consent in bariatric surgery. Surgeons should be alert to suggestive imaging findings and ready to modify operative plans.

## Introduction

Diaphragmatic hernia is a rare malformation characterized by a defect in the diaphragm, causing abdominal organs to herniate into the chest cavity in early development [1]. Diaphragmatic hernias can be congenital or acquired, and many remain clinically silent until discovered incidentally on imaging or during abdominal or thoracic surgery [2,3]. Congenital diaphragmatic hernias are uncommon, with an estimated global prevalence of 2.3 in 10,000 individuals [1]. The majority are Bochdalek hernias, resulting from developmental failure of the posterolateral diaphragmatic closure, with nearly 90% occurring on the left side due to the protective effect of the liver on the right [2,3].

While most congenital diaphragmatic hernias present in the neonatal period with respiratory distress, a subset remains asymptomatic or presents with minimal symptoms in adulthood [2,3]. Diaphragmatic hernias can be asymptomatic despite the growing hernia sac, remaining undiagnosed until they are discovered incidentally [4,5]. Various imaging modalities are used to diagnose diaphragmatic hernia, including chest radiographs, ultrasonography, computed tomography (CT), and magnetic resonance imaging, although surgical exploration is often necessary to confirm the diagnosis in patients with equivocal imaging results [5]. 

The increasing use of laparoscopic surgery has led to more frequent discovery of incidental intra-abdominal pathologies [6]. However, large congenital diaphragmatic hernias remain exceptionally rare findings during bariatric procedures [6-8]. This presents unique challenges for the operating surgeon, particularly regarding the scope of the procedure, patient comorbidities, and patient consent. This case report describes an incidental large left diaphragmatic hernia encountered during attempted laparoscopic sleeve gastrectomy in a morbidly obese patient.

## Case presentation

A 34-year-old male patient presented with morbid obesity (weight 120.7 kg, height 163.5 cm, BMI 45.17 kg/m²), chronic right hypochondrial pain exacerbated by fatty meals, anorexia, bilateral knee pain, back pain, and a history of failed conservative weight loss measures, including diet, exercise, and tirzepatide (Mounjaro injections), which was discontinued nine months prior to admission. He was an active smoker with no known drug allergies. His surgical history included recent management of a left foot fracture, which was treated with plaster immobilization two months earlier. 

Clinical examination revealed stable vital signs (blood pressure 138/81 mmHg, heart rate 78/minute, afebrile). Cardiovascular examination showed regular rhythm without murmurs or signs of decompensation. Respiratory examination revealed clear lung fields without wheeze or crackles, though the patient reported mild exertional dyspnea.

Laboratory investigations were unremarkable. The patient had normal complete blood count (hemoglobin 15.4 g/dL, white blood cells 9.66 × 10³/μL, platelets 276 × 10³/μL), coagulation studies (prothrombin time (PT) 14.3 seconds, international normalised ratio (INR) 1.03, partial thromboplastin time (PTT) 28.2 seconds), renal function (creatinine 0.92 mg/dL, urea 38.5 mg/dL), and liver enzymes (alanine transaminase 29 U/L, total bilirubin 0.4 mg/dL, alkaline phosphatase 57 U/L).

**Figure 1:**
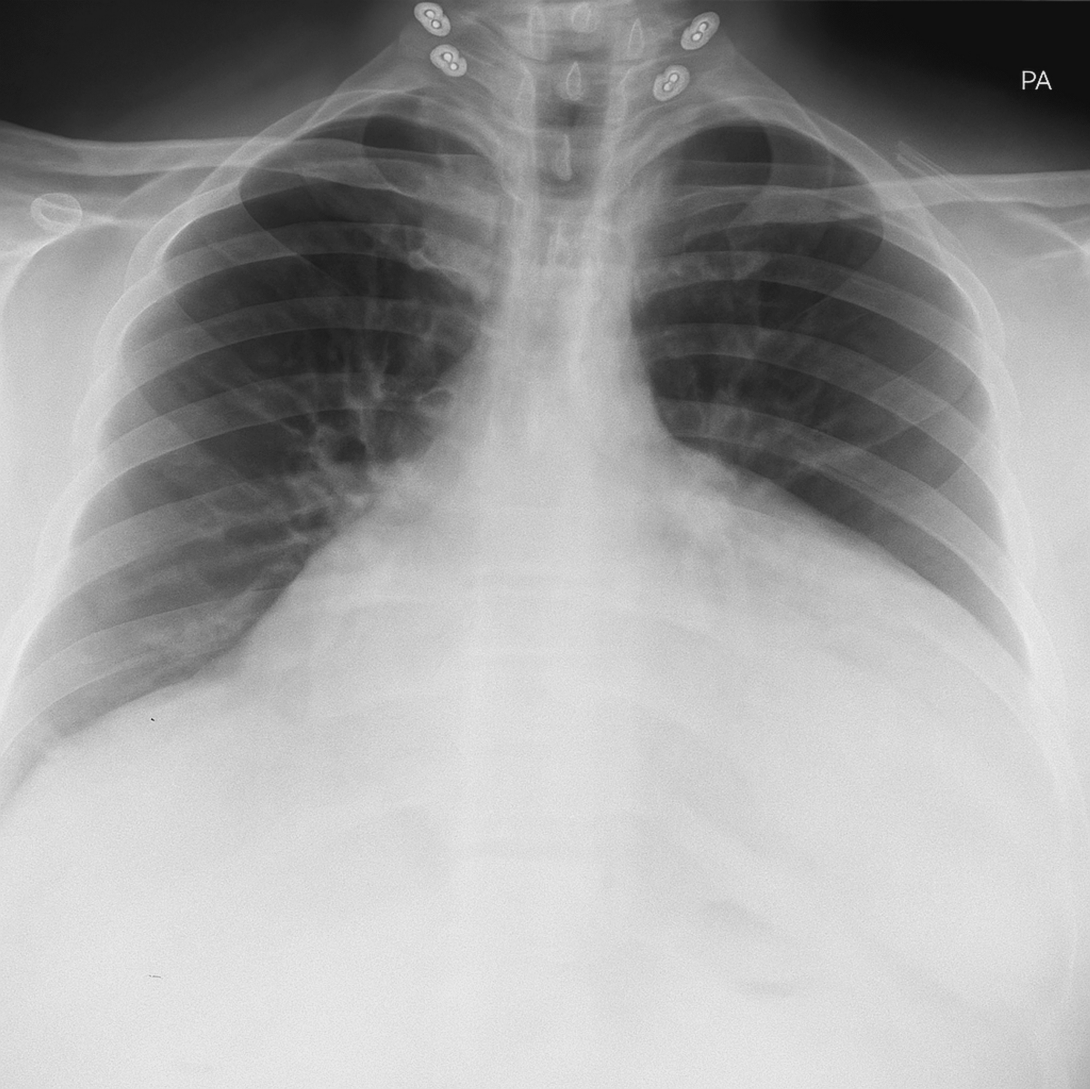
Chest X-ray showing haziness in the left lower lung field, blunting of the left costophrenic angle, and an increased cardiothoracic ratio

Abdominal ultrasonography revealed an enlarged fatty liver (16.8 cm), multiple gallbladder stones (6-9 mm), with normal common bile duct caliber. The spleen, kidneys, bladder, and prostate appeared normal with no evidence of ascites. Chest X-ray demonstrated bilateral accentuated hilar vascular shadows, left lower lung haziness, a blunted left costophrenic angle, and an increased cardiothoracic ratio. There was no overt diaphragmatic abnormality, air-fluid levels, or visible bowel gas within the thoracic cavity (Figure 1). 

The patient was scheduled for a combined laparoscopic cholecystectomy and sleeve gastrectomy under general anesthesia. After establishing pneumoperitoneum using a Veress needle, four laparoscopic ports were inserted using standard positioning for cholecystectomy. The cholecystectomy proceeded without complications. The gallbladder fundus was retracted toward the right shoulder with Hartmann's pouch displaced downward and laterally. Dissection of Calot's triangle was performed until the critical view of safety was achieved. The cystic artery and duct were identified, clipped, and divided to avoid stone movement. The gallbladder was dissected from the liver bed with good hemostasis and removed using an extraction bag. A drain was placed at the liver bed.

**Figure 2:**
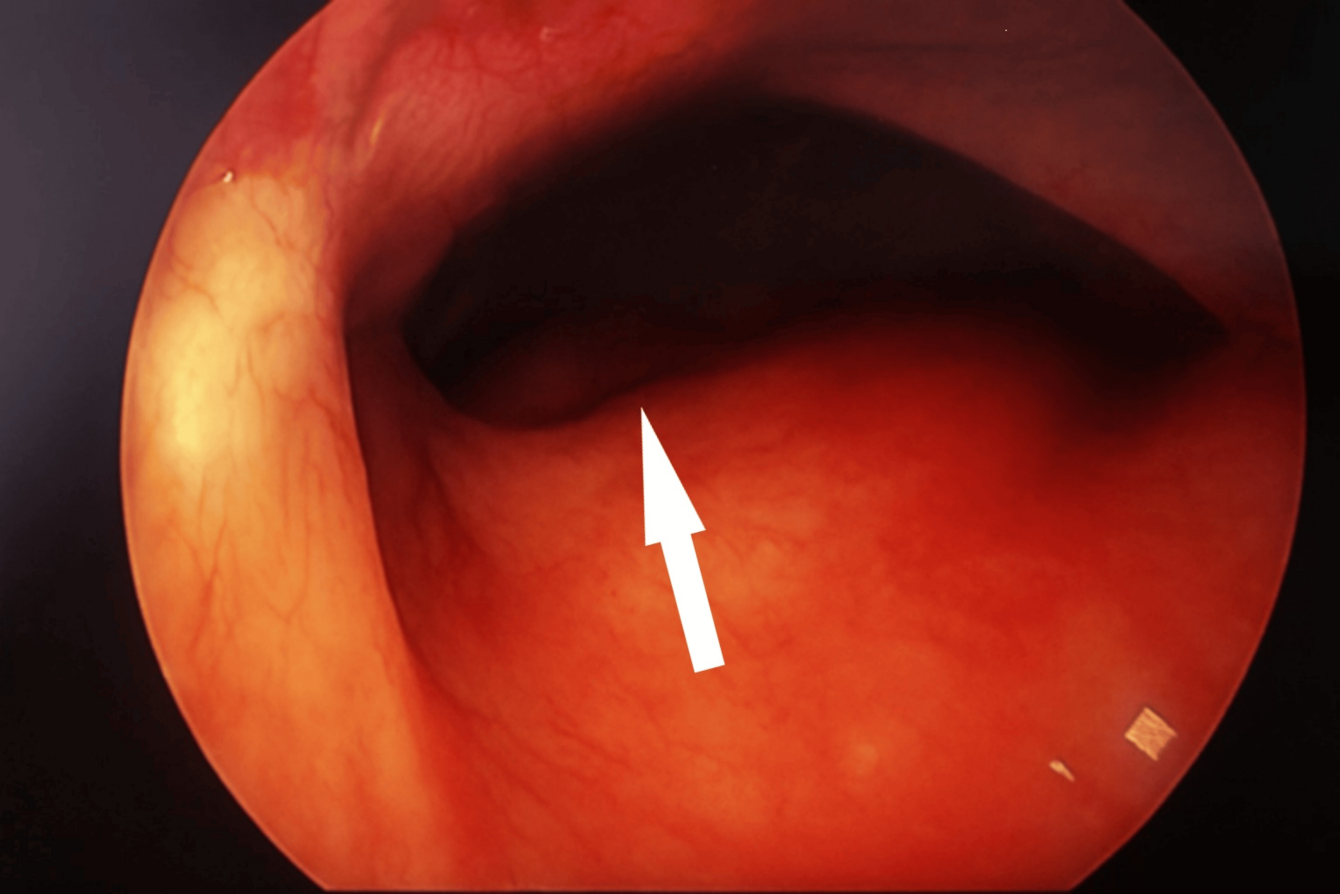
Laparoscopic view showing a diaphragmatic hernia defect (arrow)

When repositioning for the sleeve gastrectomy, an unexpected finding was discovered: a large left-sided diaphragmatic hernia (Figure 2). The defect appeared to be located in the posterolateral aspect of the left diaphragm, with a large opening allowing herniation of multiple bowel segments into the thoracic cavity. Based on the location and characteristics, this was consistent with a congenital Bochdalek hernia. Given that the patient had not been specifically consented for diaphragmatic hernia repair, the sleeve gastrectomy was aborted upon discovering the diaphragmatic hernia. The procedure concluded without complications or significant blood loss. Histopathology of the gallbladder specimen confirmed the presence of chronic calculous cholecystitis.

The patient's immediate postoperative course was uncomplicated. He was managed with standard postoperative protocols and received analgesics (paracetamol and lornoxicam), antibiotics (cefazolin and metronidazole), proton pump inhibitors (omeprazole), laxatives (Movicol), and prophylactic thromboprophylaxis (enoxaparin). Recovery was uneventful. He remained hemodynamically stable with normal vital signs and was discharged on oral medications with follow-up arrangements. The final diagnosis included morbid obesity, chronic cholecystitis, and unrepaired incidental diaphragmatic hernia, with a pending management plan.

## Discussion

Incidental diaphragmatic hernias during bariatric surgery are uncommonly reported in a few case reports, with most reported cases involving Bochdalek and Morgagni hernias detected intraoperatively [7,9,10]. This report contributes to the limited literature on diaphragmatic hernias diagnosed in adults, stressing the need for clinical awareness and timely management. Also, it emphasizes ethical decision-making, the limitations of preoperative imaging, and implications for bariatric surgical protocols. Diaphragmatic hernias can be asymptomatic despite the growing hernia sac, remaining undiagnosed until they are discovered incidentally [4,5]. 

The increasing use of laparoscopic surgery provides good visualization of the peritoneal cavity, allowing for the detection of incidental intra-abdominal pathology that may not be apparent on preoperative imaging. A recent cross-sectional study of 534 abdominal surgeries reported incidental findings in 1.1% of cases, indicating that unexpected findings during surgery represent a clinical challenge [6]. Epidemiological information about incidental findings in abdominal surgery can be helpful for the surgeon to be much more prepared for the surgery and the possible unex­pected lesions that might appear [6].

In our case, the chest X-ray showed several abnormalities that were not initially recognized as suggestive of diaphragmatic pathology. The literature indicates that preoperative diagnosis of adult diaphragmatic hernia can be challenging due to subtle or nonspecific imaging findings. While a plain chest X-ray is often the first imaging study, previous studies showed that the diagnosis may be missed in more than 20% of cases if a CT was not performed [11-14]. The diagnostic challenge is compounded by the fact that many adult congenital diaphragmatic hernias remain asymptomatic or present with vague symptoms that do not prompt specific imaging evaluation [10,15].

The decision to proceed with concurrent repair versus aborting the primary procedure requires careful consideration of multiple factors, including patient comorbidities, operative duration, and, most importantly, the extent of informed consent. The decision to abort a laparoscopic sleeve gastrectomy highlights ethical challenges, since proceeding without consent risks medicolegal issues.

From a patient safety perspective, aborting the laparoscopic sleeve gastrectomy upon discovering a large left diaphragmatic hernia was justified due to lack of consent, as well as the increased risks caused by the patient’s morbid obesity and the hernia’s significant size, containing colon and small bowel. Although the literature is scarce, previous case reports support the safety of concurrent diaphragmatic hernia repair during bariatric surgery [7,8,16]. However, concurrent repair shows variable outcomes with a higher risk of reoperation and readmission [17]. A previous review reported that only a few studies described the efficacy and safety of concomitant repair during bariatric surgery, but acknowledged the lack of guidelines and large-scale studies addressing this issue, and suggested that surgeons thoroughly assess their patients’ overall clinical picture [7].

## Conclusions

This case demonstrates the complex challenges involved in managing incidental congenital diaphragmatic hernias discovered during laparoscopic surgery, specifically sleeve gastrectomy. Key learning points include the importance of systematic diaphragmatic evaluation during laparoscopic procedures, recognition that imaging may not always be conclusive for diaphragmatic pathology, and the value of clear institutional policies regarding the scope of informed consent and management of incidental discoveries. Enhanced preoperative imaging and consent processes may mitigate such dilemmas, improving patient safety and outcomes.
